# A discriminative method for protein remote homology detection and fold recognition combining Top-*n*-grams and latent semantic analysis

**DOI:** 10.1186/1471-2105-9-510

**Published:** 2008-12-01

**Authors:** Bin Liu, Xiaolong Wang, Lei Lin, Qiwen Dong, Xuan Wang

**Affiliations:** 1 Harbin Institute of Technology Shenzhen Graduate School, Shenzhen, PR China; 2 School of Computer Science and Technology, Harbin Institute of Technology, Harbin, PR China

## Abstract

**Background:**

Protein remote homology detection and fold recognition are central problems in bioinformatics. Currently, discriminative methods based on support vector machine (SVM) are the most effective and accurate methods for solving these problems. A key step to improve the performance of the SVM-based methods is to find a suitable representation of protein sequences.

**Results:**

In this paper, a novel building block of proteins called Top-*n*-grams is presented, which contains the evolutionary information extracted from the protein sequence frequency profiles. The protein sequence frequency profiles are calculated from the multiple sequence alignments outputted by PSI-BLAST and converted into Top-*n*-grams. The protein sequences are transformed into fixed-dimension feature vectors by the occurrence times of each Top-*n*-gram. The training vectors are evaluated by SVM to train classifiers which are then used to classify the test protein sequences. We demonstrate that the prediction performance of remote homology detection and fold recognition can be improved by combining Top-*n*-grams and latent semantic analysis (LSA), which is an efficient feature extraction technique from natural language processing. When tested on superfamily and fold benchmarks, the method combining Top-*n*-grams and LSA gives significantly better results compared to related methods.

**Conclusion:**

The method based on Top-*n*-grams significantly outperforms the methods based on many other building blocks including N-grams, patterns, motifs and binary profiles. Therefore, Top-*n*-gram is a good building block of the protein sequences and can be widely used in many tasks of the computational biology, such as the sequence alignment, the prediction of domain boundary, the designation of knowledge-based potentials and the prediction of protein binding sites.

## Background

Protein homology detection is one of the most intensively researched problems in bioinformatics. Researchers are increasingly depending on computational techniques to classify proteins into functional or structural classes by means of homologies. Most methods can detect homologies at high levels of sequence similarity, while accurately detecting homologies at low levels of sequence similarity (remote homology detection) is still a challenging problem.

Many powerful methods and algorithms have been proposed to address the remote homology detection and fold recognition problems. Some methods are based on the pairwise similarities between protein sequences. Smith-Waterman dynamic programming algorithm [1] finds an optimal score for similarity according to a predefined objective function. RANKPROP [2] relies upon a precomputed network of pairwise protein similarities. Some heuristic algorithms, such as BLAST [3] and FASTA [4] trade reduced accuracy for improved efficiency. These methods do not perform well for remote homology detection, because the alignment score falls into a twilight zone when the protein sequences similarity is below 35% at the amino acid level [5]. Later methods challenge this problem by incorporating the family information. These methods are based on a proper representation of protein families and can be split into two groups [6]: generative models and discriminative algorithms. Generative models provide a probabilistic measure of association between a new sequence and a particular family. These methods such as profile hidden Markov model (HMM) [7] can be trained iteratively in a semi-supervised manner using both positively labeled and unlabeled samples of a particular family by pulling in close homology and adding them to the positive set [8]. The discriminative algorithms such as Support Vector Machines (SVM) [9] provide state-of-the-art performance. In contrast to generative models, the discriminative algorithms focus on learning a combination of the features that discriminate between the families. These algorithms are trained in a supervised manner using both positive and negative samples to establish a discriminative model. The performance of SVM depends on the kernel function, which measures the similarity between any pair of samples. There are two approaches for deriving the kernel function. One approach is the direct kernel, which calculates an explicit sequence similarity measure. Another approach is the feature-space-based kernel, which chooses a proper feature space, represents each sequence as a vector in that space and then inner product (or a function derived from it) between these vector-space representations is taken as a kernel for the sequences [10].

### Direct kernel

LA kernel [11] is one of the direct kernel functions. This method measures the similarity between a pair of protein sequences by taking into account all the optimal local alignment scores with gaps between all possible subsequences. Another method is SW-PSSM [10] which is derived directly from explicit similarity measures and utilizes sequence profiles. The experiment results show that this kernel is superior to previously developed schemes.

### Feature-space-based kernel

SVM-Fisher [12] is one of the early attempts with the feature-space-based kernel, This method represents each protein sequence as a vector of Fisher scores extracted from a profile hidden Markov model (HMM) for a protein family. SVM-pairwise [13] is another successful method, in which each protein sequence is represented as a vector of pairwise similarities to all protein sequences in the training set. The SVM-I-sites method [14] compares sequence profiles to the I-sites library of local structural motifs for feature extraction. Later, this method is improved by using the order and relationships of the I-site motifs [15]. SVM-BALSA [16] represents each protein sequence by a vector of Bayesian alignment scores associated with each training sample. The feature spaces in Spectrum kernel [17] consist of all short subsequence of length *k *("*k*-mers"). SVM-n-peptide [18] improves Spectrum kernel by using reduced amino acid alphabets to reduce the dimensions of the feature vectors. Mismatch kernel [19] considers a *k*-mer to be present if a sequence contains a substring which differs with the *k*-mer at most a predefined number of mismatches. Profile kernel [20] considers a *k*-mer to be present if a sequence contains a substring whose PSSM-based ungapped alignment score with the *k*-mer is above a predefined threshold. Lingner and Meinicke [6] notice the distances between *k*-mers and introduce a feature vector based on the distances between the *k*-mers. The feature vector of eMOTIF kernel [21] is based on motifs extracted with the unsupervised eMOTIF method [22] from the eBLOCKS database [23]. GPkernel [24] is another motif kernel based on discrete sequence motifs, which are evolved using genetic programming. SVM-HUSTLE [25] builds a SVM classifier for a query sequence by training on a collection of representative high-confidence training sets, recruits additional sequences and assigns a statistical measure of homology between a pair of sequences.

Multiple sequence alignments of protein sequences contain a lot of evolutionary information. This information can be obtained by analyzing the output of PSI-BLAST [26,27]. Since the protein sequence frequency profile is a richer encoding of protein sequences than the individual sequence, it is of great significance to use such evolutionary information for protein remote homology detection and fold recognition. In our previous study, we introduced a feature vector of protein sequence based on binary profiles [28] which contain the evolutionary information of the protein sequence frequency profiles. The protein sequence frequency profiles are converted into binary profiles with a probability threshold. In detail, when the frequency of a given amino acid is higher than the threshold it is converted into an integral value 1, otherwise it is converted into 0. Binary profile can be viewed as a building block of proteins. It has been successfully applied in many computational biology tasks, such as the domain boundary prediction [29], the knowledge-based mean force potentials [30] and the protein binding sites prediction [31]. Although the methods based on binary profiles give exciting results, binary profiles have several shortcomings. Firstly, the threshold which is used to convert the protein sequence frequency profiles into binary profiles is set by experience. Because there is no systematic method that could be used to optimize the threshold, there is no guarantee to find the best threshold. Secondly, binary profiles cannot discriminate the amino acids with different frequencies in the protein sequence frequency profiles. The amino acids whose frequencies are higher than the threshold are all converted into 1, which omits that these amino acids have different frequencies and different importance during evolutionary processes.

To overcome these shortcomings, in this study we present a novel building block of proteins called Top-*n*-grams to use the evolutionary information of the protein sequence frequency profiles and apply this novel building block to remote homology detection and fold recognition. The protein sequence frequency profiles calculated from the multiple sequence alignments outputted by PSI-BLAST [26] are converted into Top-*n*-grams by combining the *n *most frequent amino acids in each amino acid frequency profile. The protein sequences are transformed into fixed-dimension feature vectors by the occurrence times of each Top-*n*-gram and then the corresponding vectors are inputted to SVM. In our previous studies [28,32], we applied LSA [33] to protein remote homology detection. Several basic building blocks have been investigated as the "words" of "protein sequence language", including N-grams [17], patterns [34], motifs [21] and binary profiles [28]. Here, we also demonstrate that the use of LSA on Top-*n*-grams can improve the prediction performance of both remote homology detection and fold recognition.

## Results and discussion

### Comparative results of various methods

We compare our method with many other methods. Table 1 and Table 2 compare the performance of the method proposed in this paper against that achieved by a number of previously developed methods for remote homology detection and fold recognition. In the case of remote homology detection, the performance is compared against PSI-BLAST [26], pairwise [13], LA [11] and four building-block-based methods (N-gram [17], pattern [34], motif [21] and binary profiles [28]). For detailed setup procedures of these methods, please refer to our previous studies [28,32]. We also include the results of two state-of-the-art methods, Profile [20] and SW-PSSM [10]. In the case of fold recognition, we include results of LA [11], PSI-BLAST [26], pairwise [13], GPkernel [24], Mismatch [19], eMOTIF [21], binary profiles [28], Profile [20] and SW-PSSM [10].

**Table 1:** Comparison against different methods on SCOP 1.53 superfamily benchmark

Average ROC and ROC50 scores			
Methods	ROC	ROC50	Source

SVM-Top-*n*-gram			
*n *= 1	0.9069	0.696	
*n *= 2	0.9230	0.713	
*n *= 3	0.9073	0.653	
SVM-Top-*n*-gram-combine	0.9329	0.763	
SVM-Bprofile(*Ph *= 0.13)	0.9032	0.681	[28]
SVM-Ngram	0.7914	0.584	[32]
SVM-Pattern	0.8354	0.589	[32]
SVM-Motif	0.8136	0.616	[32]
			
SVM-Top-*n*-gram-combine-LSA	0.9390	0.767	
SVM-Bprofile-LSA(*Ph *= 0.13)	0.9210	0.698	[28]
SVM-Ngram-LSA	0.8595	0.628	[32]
SVM-Pattern-LSA	0.8789	0.626	[32]
SVM-Motif-LSA	0.8592	0.628	[32]
			
PSI-BLAST	0.6754	0.330	[32]
SVM-Pairwise	0.8960	0.464	[11]
SVM-LA(*β *= 0.5)	0.9250	0.649	[11]
Profile(5,7.5)	0.9800	0.794	[10]
SW-PSSM(3.0,0.750,1.50)	0.9820	0.904	[10]

SVM-Ngram, SVM-Pattern, SVM-Motif, SVM-Bprofile and SVM-Top-*n*-gram refer to the methods based on the five building blocks: N-grams, patterns, motifs, binary profiles and Top-*n*-grams respectively. The methods with combine suffix refer to the methods combining Top-1-grams and Top-2-grams. The methods with LSA suffix refer to the corresponding methods after latent semantic analysis. Source is the sources of results.

**Table 2:** Comparison against different methods on SCOP 1.67 fold benchmark

Average ROC and ROC50 scores			
Methods	ROC	ROC50	Source

SVM-Top-*n*-gram			
*n *= 1	0.7778	0.649	
*n *= 2	0.8130	0.642	
*n *= 3	0.7960	0.628	
SVM-Top-*n*-gram-combine	0.8180	0.677	
SVM-Bprofile(*Ph *= 0.11)	0.8042	0.644	
			
SVM-Top-*n*-gram-combine-LSA	0.8535	0.694	
SVM-Bprofile-LSA(*Ph *= 0.11)	0.8233	0.658	
			
PSI-BLAST	0.5010	0.010	[24]
SVM-Pairwise	0.7240	0.359	[24]
SVM-LA	0.8340	0.504	[24]
Gpkernel	0.8440	0.514	[24]
Mismatch	0.8140	0.467	[24]
eMOTIF	0.6980	0.308	[24]

SVM-Bprofile and SVM-Top-*n*-gram refer to the methods based on the two building blocks: binary profiles and Top-*n*-grams respectively. The methods with combine suffix refer to the methods combining Top-1-grams and Top-2-grams. The methods with LSA suffix refer to the corresponding methods after latent semantic analysis. The results of the methods based on binary profiles were obtained by running the implementation of our previous work.

### The influence of *n *on remote homology and fold recognition

Top-*n*-grams are generated by combining the *n *most frequent amino acids in the amino acid frequency profiles (see method section for details). Here, we investigate the influence of *n *on the prediction performance. As shown in Table 1 and Table 2, in terms of the ROC50 scores, the rates indicate that our method performs well for *n *= 1 and *n *= 2 and with a slight decrease of the ROC50 score for *n *= 3. The third most frequent amino acids in the amino acid frequency profiles are less likely to occur in the specific sequence positions during evolutionary processes, which might be the reason for the decrease of the prediction performance for *n *= 3.

**Figure 1:**
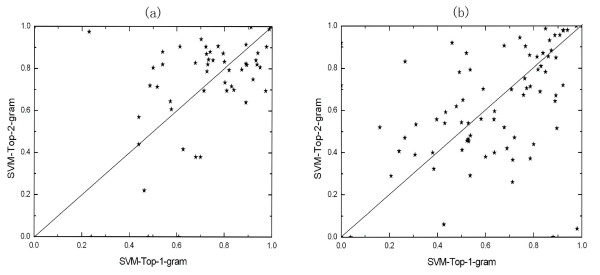
**Top-1-gram's ROC50 scores plotted against those of Top-2-gram on SCOP 1.53 superfamily (a) and SCOP 1.67 fold (b)benchmarks**. Identical ROC50 scores of the two methods would fall on the diagonal line.

We plot Top-1-grams's ROC50 scores against Top-2-grams's ROC50 scores on all the test sets for the SCOP 1.53 superfamily benchmark and SCOP 1.67 fold benchmark (Figure 1). This figure show that the two building blocks are complementary, so they can be combined to create a new building block of proteins to solve remote homology detection and fold recognition problems. In detail, the protein sequences are transformed into fixed-dimension feature vectors by the occurrence times of each Top-*n*-gram in Top-1-grams and Top-2-grams. Therefore, these vectors are the combinations of the corresponding vectors of the method based on Top-1-grams and the ones of the method based on Top-2-grams. The dimension of the combined feature vector is 420 (20+400). Compared with the best ROC50 scores achieved by the method based on single building block, the ROC50 scores of the method based on the combined building block are 5 percent and 2.8 percent higher for remote homology detection and fold recognition, respectively.

### LSA can improve the performance of building-block-based methods for both remote homology and fold recognition problems

**Figure 2:**
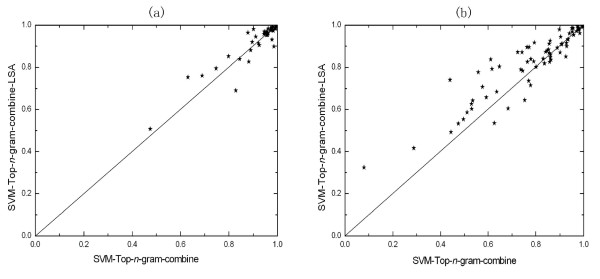
**Comparison of the method with LSA and without LSA on SCOP 1.53 superfamily (a) and SCOP 1.67 fold benchmarks (b)**. The coordinate of each point in each sub-figure is the ROC score for one test set, obtained by the two methods labelled near the axis.

The latent semantic analysis [32] is used on Top-*n*-grams to remove noise and compress data. Figure 2(a) shows the family-by-family comparison of the ROC scores between the method with LSA and without LSA when Top-*n*-grams are taken as the basic building blocks on SCOP 1.53 superfamily benchmark. Each point on the figure corresponds to one of the tested families. When the families are in the left-upper area, it means that the method labelled by y-axis outperforms the method labelled by x-axis on this family. Figure 2(b) shows the superfamiy-by-superfamily comparison of the ROC scores on SCOP 1.67 fold benchmark. Obviously, when Top-*n*-grams are taken as the building blocks, the method with LSA outperforms the method without LSA for both remote homology detection and fold recognition. As shown in Table 1, when LSA is applied, performance improvement is also observed for the methods using N-grams, patterns, motifs and binary profiles as the building blocks.

### The method based on Top-*n*-gram significantly outperforms the other building-block-based methods and most existing methods

**Figure 3:**
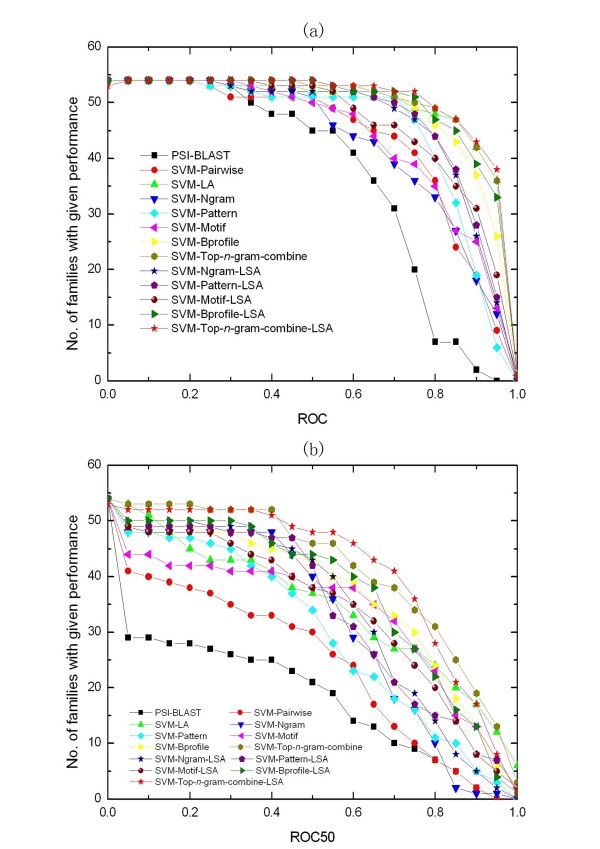
**Comparison of some common methods for remote homology detection on SCOP 1.53 superfamily benchmark**. The graphs plot the total number of families for which a given method exceeds a ROC (a) and ROC50 (b) score threshold.

**Figure 4:**
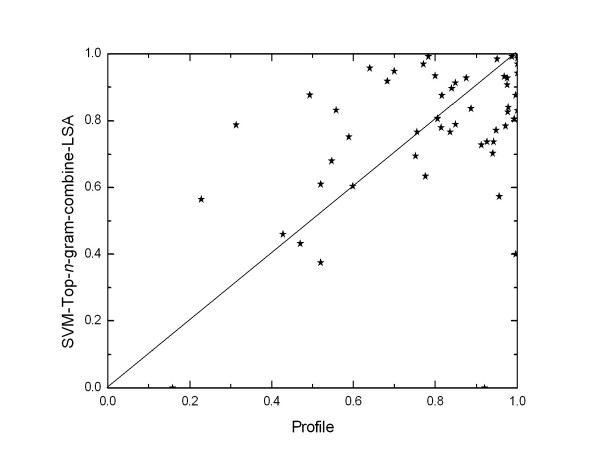
**Top-*n*-gram-combine-LSA's ROC50 scores plotted against those of Profile on SCOP 1.53 superfamily benchmark**. Identical ROC50 scores of the two methods would fall on the diagonal line.

Our method significantly outperforms the other building-block-based methods and most existing methods, except Profile and SW-PSSM. Figure 3 shows the relative performance of the compared methods on the SCOP 1.53 superfamily benchmark. SVM-Top-*n*-gram-combine-LSA significantly outperforms the four building-block-based methods in terms of ROC scores (*p*-values of 3e-9, 2e-5, 1e-5 and 0.004 – Wilcoxon signed-rank test with Bonferroni correction – for SVM-Ngram-LSA, SVM-Pattern-LSA, SVM-Motif-LSA and SVM-Bprofile-LSA) and ROC50 scores (*p*-values of 4e-6, 0.0006, 4e-6 and 0.009). SVM-Top-*n*-gram-combine-LSA also has significantly higher ROC scores than PSI-BLAST and SVM-Pairwise (*p *= 2e-9 and 8e-5) and significantly higher ROC50 scores than PSI-BLAST, SVM-Pairwise and SVM-LA (*p *= 4e-8, 3e-8 and 0.02), but SVM-Top-*n*-gram-combine-LSA and SVM-LA's ROC scores are not significantly different (*p *= 0.9). In terms of ROC scores Profile is significantly better than SVM-Top-*n*-gram-combine-LSA (*p *= 7e-6), but Profile's ROC50 scores are not significantly better than those of SVM-Top-*n*-gram-combine-LSA (*p *= 0.4). SW-PSSM is the best method in terms of ROC scores (*p *= 3e-6) and ROC50 scores (*p *= 2e-5). By plotting SVM-Top-*n*-gram-combine-LSA's ROC50 scores against Profile's ROC50 scores on all test sets (Figure 4), we find that the two methods are complementary. This result is not surprising, because the two methods depend on distinct features. Maybe the two methods can be combined to create an improved method for protein remote homology detection by using the method that is recently proposed by Damoulas and Girolami [35].

**Figure 5:**
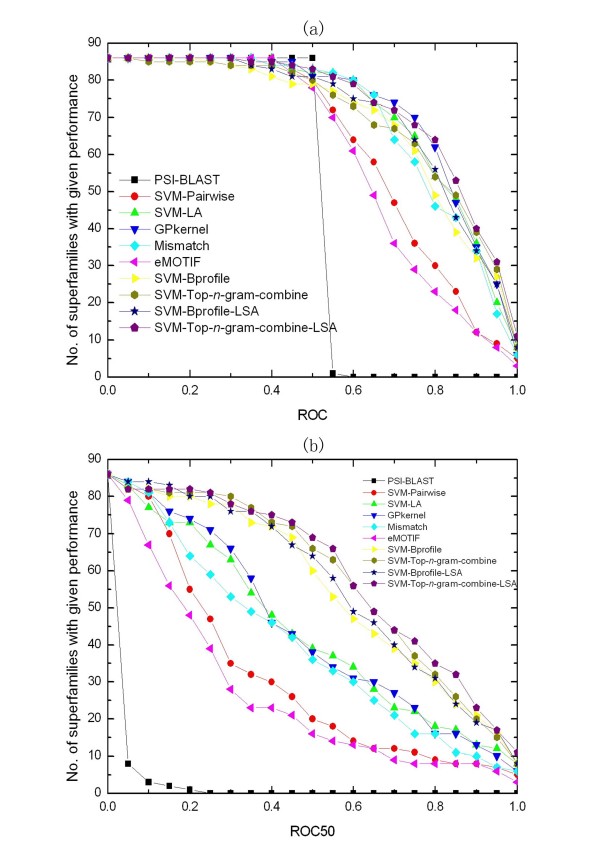
**Comparison of some common methods for fold recognition on SCOP 1.67 fold benchmark**. The graphs plot the total number of superfamilies for which a given method exceeds a ROC (a) and ROC50 (b) score threshold.

Figure 5 shows the relative performance of the compared methods on the SCOP 1.67 fold benchmark. SVM-Top-*n*-gram-combine-LSA significantly outperforms PSI-BLAST, SVM-Pairwise, Mismatch, eMOTIF and SVM-Bprofile-LSA in terms of ROC scores (*p *= 2e-15, 3e-10, 0.0001, 1e-11 and 0.001) and ROC50 scores (*p *= 2e-15, 4e-13, 2e-11, 3e-14 and 0.01). SVM-Top-*n*-gram-combine-LSA also has significantly higher ROC50 scores than SVM-LA and Gpkernel (*p *= 8e-8 and 2e-10), but their ROC scores are not significantly different (*p *= 0.08 and 0.4). Because the SCOP 1.67 fold benchmark is a recently established benchmark, the results of Profile and SW-PSSM are not reported. Furthermore, both the two methods contain several parameters which need to be optimized for different dataset. For fair comparisons, we benchmark our method on Rangwala and Karypis's SCOP 1.53 fold benchmark [10,36]. Table 3 summarizes SVM-Top-*n*-gram-LSA's average ROC score and ROC50 score on the 23 test sets and compares the averages to the averages of Profile, SW-PSSM and SVM-Bprofile-LSA. The results confirm SVM-Top-*n*-gram-LSA has better performance than SVM-Bprofile-LSA (*p *= 0.3 and *p *= 0.05 for ROC and ROC50). In terms of ROC scores (*p *= 5e-4) and ROC50 scores (*p *= 0.003), the differences between SVM-Top-*n*-gram-LSA and Profile are significant. SW-PSSM is the best method (*p *= 2e-4 and 0.04 for ROC and ROC50).

**Table 3:** Comparison against different methods on SCOP 1.53 fold benchmark

Average ROC and ROC50 scores			
Methods	ROC	ROC50	Source

SVM-Top-*n*-gram			
*n *= 1	0.7309	0.319	
*n *= 2	0.7929	0.490	
*n *= 3	0.7740	0.314	
SVM-Bprofile(*Ph *= 0.15)	0.7849	0.352	
			
SVM-Top-*n*-gram – LSA(*n *= 2)	0.8121	0.552	
SVM-Bprofile-LSA(*Ph *= 0.15)	0.8047	0.419	
			
Profile(5,7.5)	0.9240	0.314	[10]
SW-PSSM(3.0,0.450,2.0)	0.9360	0.571	[10]

SVM-Bprofile and SVM-Top-*n*-gram refer to the methods based on the two building blocks: binary profiles and Top-*n*-grams respectively. The methods with LSA suffix refer to the corresponding methods after latent semantic analysis. Source is the sources of results. The results of the methods based on binary profiles were obtained by running the implementation of our previous work.

### Computational efficiency

One important aspect of any homology detection method is its computational efficiency. In this regard, SVM-Top-*n*-gram-combine-LSA is comparable with Profile and more efficient than SVM-Bprofile-LSA, SVM-Pattern-LSA, SVM-Motif-LSA, SVM-pairwise, SVM-LA and SW-PSSM, but a little worse than SVM-Ngram-LSA, PSI-BLAST and the method without LSA.

Table 4 summarizes the time complexities of different SVM-based methods. Any SVM-based method includes a vectorization step and an optimization step [37]. The time complexity of the vectorization step of building-block-based method is O (*nml*) [32], where *n *is the number of training samples, *m *is the total number of words and *l *is the length of the longest training sequence. The optimization step of building-block-based method is O (*n*^2^*m*). The time complexities of pre-processing steps for different building-block-based methods are different. For the method based on Top-*n*-grams, it roughly takes O (*n*^2^*l*) to generate protein sequence frequency profiles by running PSI-BLAST. This running time is also needed for the method based on binary profiles to generate binary profiles. For the method based on N-grams, the time complexity during pre-processing step is O (*nl*). Patterns are extracted by TEIRESIAS [38], which roughly takes O (*nl*log*nl*+*n*^2^*l*^2^*m*) [39]. The running time of running MEME [40] to discover motifs is O (*n*^2^*l*^2^*W*) [41], where *W *is the width of the motif. In the current situation, when *W *= 10, the time complexity of the method based on motifs during pre-processing step is approximately equal to O (*n*^2^*l*^2^). For the LSA method, it has an additional SVD process, which roughly takes O (*nmt*) [42], where *t *is the minimum of *n *and *m*. The optimization step of LSA method is O (*n*^2^*R*), where *R *is the length of the latent semantic feature vector. Although N-grams have more words than Top-*n*-grams (Table 5), the time complexity of pre-processing step for SVM-Ngram-LSA is much lower than the time complexity of pre-processing step for SVM-Top-*n*-gram-combine-LSA (*nl *<<*n*^2^*l*). Therefore, the total running time of SVM-Ngram-LSA is lower than that of SVM-Top-*n*-gram-combine-LSA. Compared with the other three building-block-based methods, because Top-*n*-grams have the least words (Table 5), the method based on Top-*n*-grams has the lowest running time. The vectorization step of SVM-pairwise takes a running time of O (*n*^2^*l*^2^) and the optimization step is O (*n*^3^), which lead to a total time complexity of O (*n*^2^*l*^2^) + O (*n*^3^). LA-kernel and SVM-pairwise has the same time complexity [11], so the running time of SVM-Top-*n*-gram-combine-LSA is lower than SVM-pairwise and SVM-LA. The time complexity of pre-processing step for Profile is the same as the time complexity of pre-processing step for SVM-Top-*n*-gram-combine-LSA and the calculation of Profile kernel roughly takes O (*n*^2^*k*^*p*+1^20^*p*^*l*) [20], where *k *is the length of short subsequence and *p *is the number of mismatches in the short subsequence. In the current situation, when *k *= 5, *p *= 1 the time complexity of calculation of Profile kernel is approximately equal to O (*n*^2^*l*). Thus, the running time of SVM-Top-*n*-gram-combine-LSA is comparable with Profile. The time complexity of the pre-processing step for SW-PSSM is the same as the time complexity of the pre-processing step for SVM-Top-*n*-gram-combine-LSA and the time complexity of calculation of SW-PSSM kernel is O (*n*^2^*l*^2^). In practice, since *m *<<*nl*, the running time ofSVM-Top-*n*-gram-combine-LSA is lower than SW-PSSM. PSI-BLAST has the lowest running time O (*nN*), where *N *is the size of the database. In the current situation, *N *is approximately equal to *nl*.

**Table 4:** Time complexities of different SVM-based methods for remote homology detection

Methods	Time complexities
SVM-Top-*n*-gram-combine	O (*n*^2^*l*) + O (*nml*) + O (*n*^2^*m*)
SVM-Bprofile	O (*n*^2^*l*) + O (*nml*) + O (*n*^2^*m*)
SVM-Ngram	O (*nl*) + O (*nml*) + O (*n*^2^*m*)
SVM-Pattern	O (*nl*log*nl*+*n*^2^*l*^2^*m*) + O (*nml*) + O (*n*^2^*m*)
SVM-Motif	O (*n*^2^*l*^2^) + O (*nml*) + O (*n*^2^*m*)
	
SVM-Top-*n*-gram-combine-LSA	O (*n*^2^*l*) + O (*nml*) + O (*nmt*) + O (*n*^2^*R*)
SVM-Bprofile-LSA	O (*n*^2^*l*) + O (*nml*) + O (*nmt*) + O (*n*^2^*R*)
SVM-Ngram-LSA	O (*nl*) + O (*nml*) + O (*nmt*) + O (*n*^2^*R*)
SVM-Pattern-LSA	O (*nl*log*nl*+*n*^2^*l*^2^*m*) + O (*nml*) + O (*nmt*) + O (*n*^2^*R*)
SVM-Motif-LSA	O (*n*^2^*l*^2^) + O (*nml*) + O (*nmt*) + O (*n*^2^*R*)
	
SVM-Pairwise	O (*n*^2^*l*^2^) + O (*n*^3^)
SVM-LA	O (*n*^2^*l*^2^) + O (*n*^3^)
Profile	O (*n*^2^*l*) + O (*n*^2^*l*)
SW-PSSM	O (*n*^2^*l*) + O (*n*^2^*l*^2^)

Where *n *is the number of training samples, *l *is the length of the longest training sequence, *m *is the total number of the words of each building block, *t *is the minimum of *n *and *m *and *R *is the length of the latent semantic feature vector.

**Table 5:** Numbers of "words" of the five building blocks for remote homology detection

Building blocks	N-grams	patterns	motifs	binary profiles	Top-*n*-gram-combine
Numbers of "words"	8000	8000	3231	1087	420

The combine suffix refers to Top-*n*-grams combining Top-1-grams and Top-2-grams. N-grams are the set of all possible subsequences of a fixed length 3, so the total words of N-grams are 8000 (20^3^) [32]. Patterns are extracted by TEIRESIAS [38] and totally 71009 patterns are extracted [32]. Through *χ*^2 ^selection [34], 8000 patterns are selected as the characteristic words [32]. The MEME/MAST system [40] is used to discover motifs and search databases. Totally, 3231 motifs are extracted [32]. The optimized probability threshold 0.13 is used to convert the protein sequence frequency profiles into binary profiles and 1087 words are obtained [28]. Top-1-grams and Top-2-grams have 20 and 400 words respectively, so the total words of Top-*n*-gram-combine are 420 (20+400).

### Correlations between Top-*n*-grams and protein families

One of the main advantages of our method is the possibility to analyze the correlations between Top-*n*-grams and protein families and reveal biologically relevant properties of the underlying protein families.

**Figure 6:**
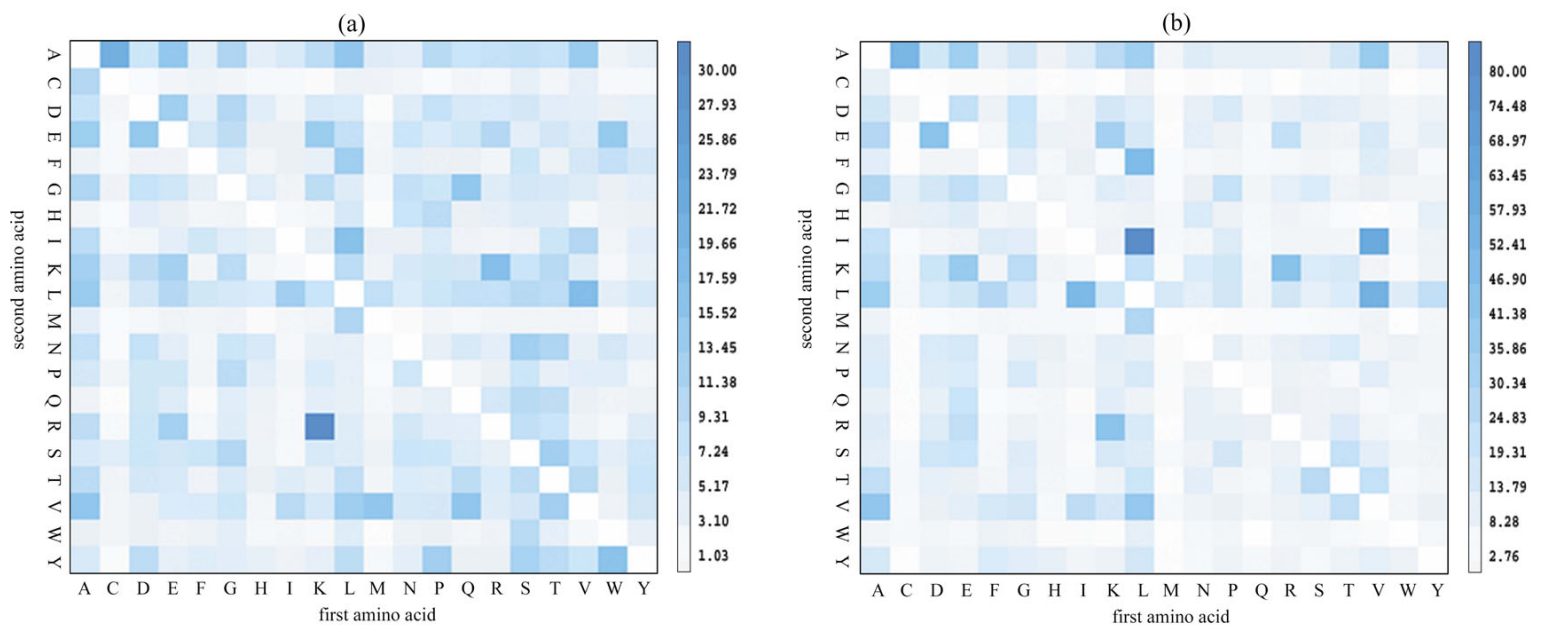
**The average chi-square score of each Top-2-gram in all tested families and superfamilies on SCOP 1.53 superfamily benchmark (a) and SCOP 1.53 fold benchmark (b)**. The amino acids are identified by their one-letter code. The amino acids labelled by x-axis and y-axis indicate the first amino acids and the second amino acids in Top-2-grams, respectively. The adjacent colour bar shows the mapping of the average chi-square scores.

**Figure 7:**
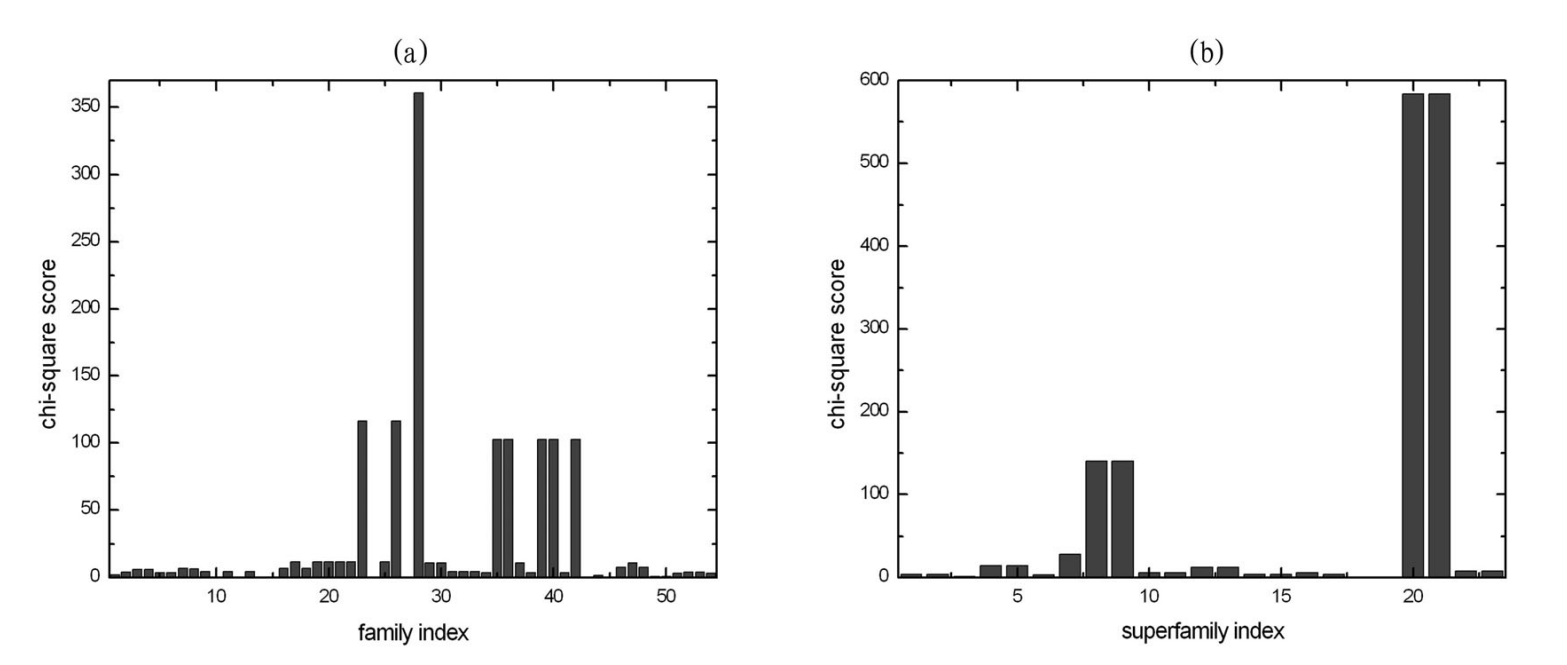
**The distribution of the chi-square scores of "KR" over different tested families on SCOP 1.53 superfamily benchmark (a) and the distribution of the chi-square scores of "LI" over different tested superfamilies on SCOP 1.53 fold benchmark (b)**. "KR" is the Top-2-gram with the highest average chi-square score (32.0716) on SCOP 1.53 superfamily benchmark and "LI" is the Top-2-gram with the highest average chi-square score (81.3576) on SCOP 1.53 fold benchmark.

In document classification field, the chi-square algorithm is one of the most effective feature selection methods, which is able to select the most discriminative features by their average chi-square scores [43]. According to the above results, Top-2-grams show highly discriminative power. Therefore, the chi-square algorithm is used to measure the correlations between Top-2-grams and protein families and can be compared to the chi-square distribution with one degree of freedom to judge extremeness (see method section for details). The average chi-square score of each Top-2-gram in all the tested families on SCOP 1.53 superfamily benchmark is shown in figure 6(a) and the average chi-square score of each Top-2-gram in all the tested superfamilies on SCOP 1.53 fold benchmark is shown in figure 6(b). According to the darkest spots in the figures, Top-2-gram "KR" has the highest score on superfamily benchmark and Top-2-grams "LI" has the highest score on fold benchmark. Therefore, these two Top-2-grams are selected for further analysis. Figure 7(a) shows the chi-square scores of "KR" in all the 54 tested families on SCOP 1.53 superfamily benchmark. As shown in this figure, "KR" is highly correlated with family 28 (SCOP ID: 7.3.10.1), correlated with family 23, 26 (SCOP ID: 2.28.1.1, 2.28.1.3) and also correlated with family 35, 36, 39, 40, 42 (SCOP ID: 2.1.1.1, 2.1.1.2, 2.1.1.3, 2.1.1.4, 2.1.1.5). The test statistics for "KR" with some other families are above the threshold for statistical significance (*p *= 0.05), but these scores are considerably lower than the ones listed above. The results indicate that "KR" is highly correlated with specific families and biologically relevant families tend to having similar chi-square scores, indicating "KR" is likely to contain important structural or functional information of the specific families. Similar results are also obtained for fold recognition. Figure 7(b) shows the chi-square scores of "LI" in all the 23 tested superfamilies on SCOP 1.53 fold benchmark. "LI" is highly correlated with superfamily 20 and 21 (SCOP ID: 7.3.1, 7.3.6) and also correlated with superfamily 8, 9 (SCOP ID: 2.32.2, 2.32.3), indicating the importance of "LI" for these biologically relevant superfamilies.

## Conclusion

In this study, we present a novel representation of protein sequences based on Top-*n*-grams and apply the latent semantic analysis to improve the prediction performance of both protein remote homology detection and fold recognition. Top-*n*-grams make up of a novel building block of protein sequences and the analysis presented here is based on evolutionary information without using any structural information. Compared with other building-block-based methods, such as N-grams, patterns, motifs or binary profiles, additional evolutionary information is extracted from the protein sequence frequency profile. The experiment results show that this additional evolutionary information is relevant for discrimination.

Using standard classifier, the results show that the prediction performance of our method is highly competitive with state-of-the-art methods within the field of remote homology detection and fold recognition. Although Profile and SW-PSSM yield better results, it should be noted that both the two methods comprise several parameters. The performance of Profile depends on a smoothing parameter and the performance of SW-PSSM depends on three parameters: gap opening, gap extension and a continuous parameter zero-shift. The extensive use of parameters bares the risk of adapting the model to particular test set, which complicates a fair comparison of different methods and the application of the methods to different dataset, because new dataset is likely to require readjustment of these parameters [6]. Furthermore, because the performance can decrease for non-optimal values of these parameters, a time-consuming model selection process would be necessary for these methods to achieve optimal results. In contrast, homogeneity of ROC scores for Top-1-grams and Top-2-grams indicates the good generalization performance of our method, which obviates the tuning of any parameter and therefore our method does not require a time consuming optimization and avoids the risk of fitting the data to the test set.

Another advantage of our approach arises from the explicit feature space representation: the possibility to measure the correlations between Top-*n*-grams and protein families. Compared with other building-block-based methods, Top-*n*-grams significantly improve the detection performance while preserving the favourable interpretability of the building-block-based methods by an explicit feature space representation. As shown in results and discussion section, our method allows the researchers to analyze the correlations between Top-*n*-grams and protein families and reveal biologically relevant properties of the underlying protein families. In contrast, direct-kernel-based methods without explicit feature spaces, such as SW-PSSM and SVM-LA, do not provide an intuitive insight into the associated feature space for further analysis of relevant sequence features. Therefore, these kernels do not offer additional utility for researchers who are interested in finding the characteristic features of protein families.

Top-*n*-grams make up of a novel building block of protein sequences, which contains the evolutionary information extracted from the protein sequence frequency profiles. The results show that this building block has significantly higher discriminative power than many other building blocks. Because this novel building block is able to flexibly represent the proteins at sequence-level and residue-level, it can be used to investigate the whole protein sequence and the individual residue. Many applications of Top-*n*-grams are conceivable: e.g. the sequence alignment, the prediction of domain boundary, the designation of knowledge-based potentials and the prediction of protein binding sites.

## Methods

### Dataset description

#### SCOP 1.53 superfamily benchmark

We use a common superfamily benchmark [13] to evaluate the performance of our method for protein remote homology detection, which is available at . This benchmark has been used by many studies of remote homology detection methods [6,11,32], so it can provide good comparability with previous methods. The benchmark contains 54 families and 4352 proteins selected from SCOP version 1.53. These proteins are extracted from the Astral database [44] and include no pair with a sequence similarity higher than an E-value of 10^-25^. The proteins with lengths less than 30 are removed, because PSI-BLAST cannot generate profiles on short sequences. For each family, the proteins within the family are taken as positive test samples, and the proteins outside the family but within the same superfamily are taken as positive training samples. Negative samples are selected from outside of the superfamily and are separated into training and test sets.

#### SCOP 1.67 fold benchmark

A recently established fold benchmark [24] is used for protein fold recognition. The benchmark contains 3840 proteins and 86 superfamilies. The proteins extracted from SCOP version 1.67 are filtered with Astral database [44] and contain no pair with a sequence similarity more than 95%. The proteins with lengths less than 30 are removed. For each tested superfamily, there are at least 10 proteins in its positive training and test set. The proteins within one superfamily are taken as positive test samples, while the others in the same fold are taken as positive training samples. The negative test samples are selected from one random superfamily from each of the other folds and the negative training samples are selected from the remaining proteins. Because most of the proteins within a fold have a very low degree of similarity, this fold benchmark is considerably harder than the superfamily benchmark.

#### SCOP 1.53 fold benchmark

SCOP 1.53 fold benchmark [10,36] is another fold benchmark used for protein fold recognition. The benchmark contains 23 superfamilies and 4352 proteins selected from SCOP version 1.53. These proteins are extracted from the Astral database [44] and include no pair with a sequence similarity higher than an E-value of 10^-25^. Proteins with lengths less than 30 are removed. Proteins within the same superfamily are considered as positive test samples, and proteins within the same fold but outside the superfamily are considered as positive training samples. For each tested superfamilies, there are at least 10 positive training and 5 positive test samples. Negative samples are chosen from outside of the positive sequences' fold and split equally into test and training sets.

### Generation of protein sequence frequency profiles

In order to generate Top-*n*-grams, the protein sequence profiles are calculated in advance. A protein sequence frequency profile can be represented as a matrix ***M***, the dimensions of ***M ***are *L *× *N*, where *L *is the length of the protein sequence and *N *is the number of all standard amino acids, which is a constant value of 20. Each element of ***M ***is the target frequency which indicates the probability of an amino acid in a specific position of a protein sequence during evolutionary processes. The rows of ***M ***are the amino acid frequency profiles. For each row the elements add up to one. Each column of ***M ***corresponds to one of the 20 standard amino acids. The protein sequence frequency profiles are calculated from the multiple sequence alignments outputted by PSI-BLAST [26]. The parameter values of PSI-BLAST are set to default except that the number of iterations is set to 10. The database for PSI-BLAST to search against is nrdb90 database  from EBI [45]. A subset of multiple sequence alignments with sequence identity less than 98% is used to calculate the protein sequence frequency profiles. The sequence weight is assigned by the position-based sequence weight method [46]. The calculation of the target frequency is similar to that implemented in PSI-BLAST. Formula (1) is used to calculate the pseudo-count for amino acid *i*(*g*_*i*_).

(1) $$g_{i}=\sum_{j=1}^{20}f_{i}*(q_{ij}/p_{j})$$

where *f*_*i *_is the observed frequency of amino acid *i*, *p*_*j *_is the background frequency of amino acid *j*, *q*_*ij *_is the score of amino acid *i *being aligned to amino acid *j *in BLOSUM62 substitution matrix, which is the default score matrix of PSI-BLAST.

The target frequency is then calculated with the pseudo-count as:

(2) *Q*_*i *_= (*αf*_*i *_+ *βg*_*i*_)/(*α *+ *β*)

where *β *is a free parameter set to a constant value of 10 which is initially used by PSI-BLAST and *α *is the number of different amino acids in a given column minus one.

### Converting protein sequence frequency profiles intoTop-*n*-grams

In order to use the evolutionary information of the protein sequence frequency profiles, the protein sequence frequency profiles are converted into Top-*n*-grams. For each amino acid frequency profile, the frequencies of the 20 standard amino acids describe the probabilities of the corresponding amino acids appearing in the specific sequence positions. The higher the frequency is, the more likely the corresponding amino acid occurs. It is reasonable to use the *n *most frequent amino acids to represent the amino acid frequency profiles, because these *n *amino acids are most likely to occur at a given sequence position during evolutionary processes. The following details how to convert protein sequence frequency profiles into Top-*n*-grams.

For each amino acid frequency profile, the frequencies of the 20 standard amino acids are sorted in descending order, and then the *n *most frequent amino acids are selected and combined according to their frequencies. We call this combination of the *n *amino acids a Top-*n*-gram. Each Top-*n*-gram differentiates the different frequencies of the *n *amino acids by their different positions in the Top-*n*-gram. The above process is iterated until all amino acid frequency profiles in the protein sequence frequency profile are converted into Top-*n*-grams. In other words, a protein sequence can be converted into *k *Top-*n*-grams, where *k *is the length of the protein sequence.

**Figure 8:**
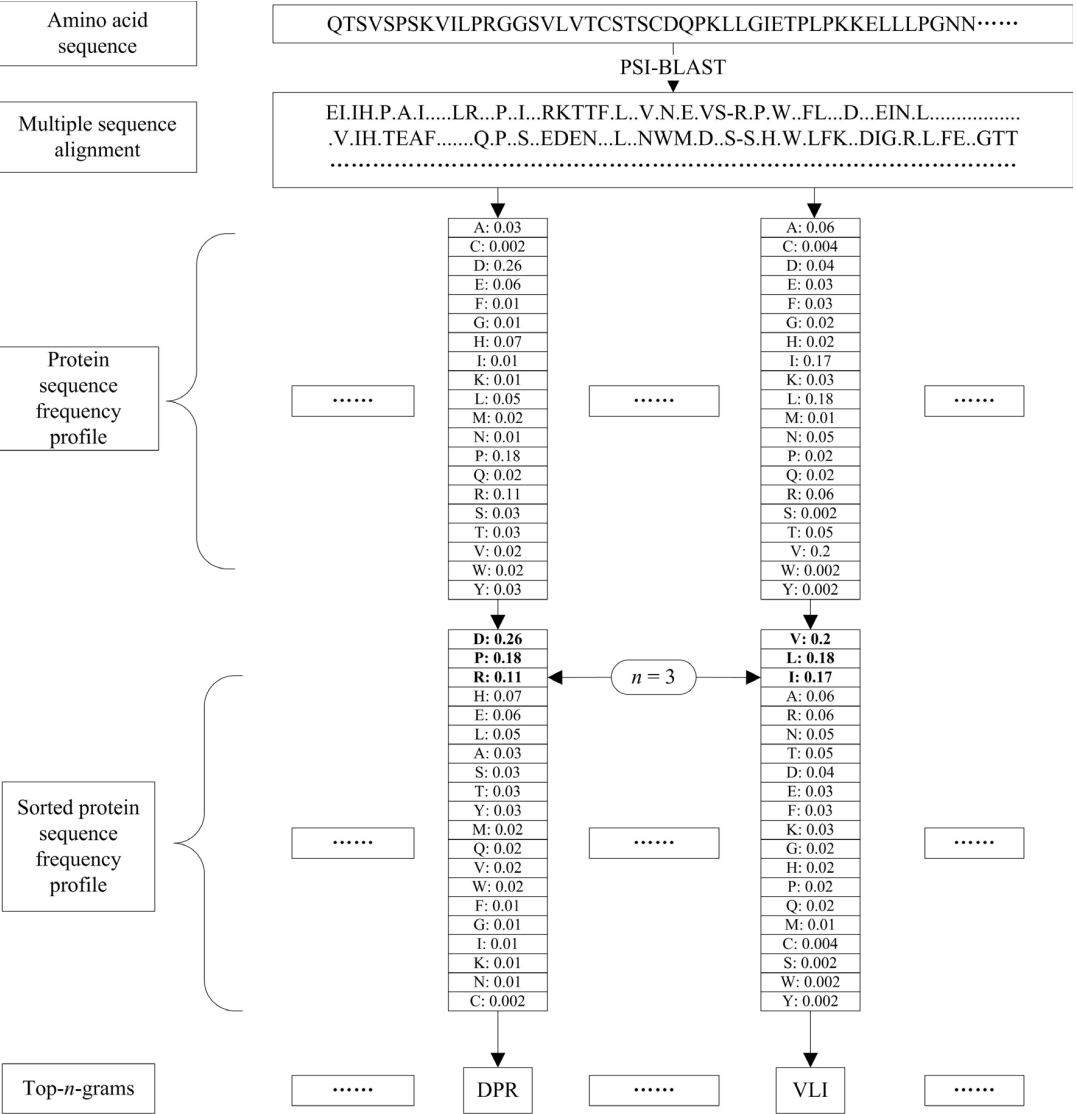
**The flowchart of generating Top-*n*-grams**. The multiple sequence alignment is obtained by PSI-BLAST. The protein sequence frequency profile is calculated from the multiple sequence alignment. The frequencies of the 20 standard amino acids in the protein sequence profile are sorted in descending order and then the sorted protein sequence frequency profile is converted to Top-*n*-grams by combining the *n *most frequent amino acids.

The process of generating and converting the protein sequence frequency profile into Top-*n*-grams is shown in Figure 8. An example of generating Top-*n*-gram (*n *= 3) "DPR" is shown in the left part of the figure. The 20 standard amino acids in the amino acid frequency profile are sorted in descending order by their frequencies and then the sorted amino acid frequency profile is converted into "DPR" by combing the 3 most frequent amino acids D, P and R. "DPR" differentiates the different frequencies of the 3 amino acids D, P and R by their different positions in "DPR". The most frequent amino acid D is in the first position of "DPR", the second most frequent amino acid P is in the second position and the third most frequent amino acid R is in the third position.

### Construction of SVM classifiers and classification

The dimension of the feature vectors of Top-*n*-grams is 20^*n*^. In this paper, Top-1-grams, Top-2-grams and Top-3-grams are investigated, the dimensions of their feature vectors are 20 (20^1^), 400 (20^2^) and 8000 (20^3^), respectively. The training proteins are transformed into fixed-dimension feature vectors by the occurrence times of each Top-*n*-gram, and then the vectors are inputted to SVM to construct the classifier for a specified class. The test proteins are vectorized in the same way as the training proteins and fed into the classifier constructed for a given class to make separation between the positive and negative samples. The SVM assigns each protein in the test set a discriminative score which indicates a predicted positive level of the protein. The proteins with discriminative scores higher than the threshold zero are classified as positive samples and the others as negative ones. The above process is iterated until each class is tested.

The focus of this study is to find a suitable representation of protein sequences. In order to exclude differences owing to particular realizations of the SVM-based learning algorithm and for best comparability with other methods, we employ the publicly available Gist SVM package version 2.1.1 . The SVM parameters are used by default of the Gist Package except that the kernel function is set as Radial Basis Function (RBF).

### Latent Semantic Analysis

Latent Semantic Analysis (LSA) is a theory and method for extracting and representing the contextual-usage meaning of words by statistical computations applied to a large corpus of text [42]. Recently, LSA was introduced in computational biology, such as protein secondary structure prediction [47] and protein remote homology detection [32]. The process of LSA is as follows:

Firstly, a word-document matrix ***W ***of co-occurrences between words and documents is constructed. The elements of ***W ***indicate the number of times each word appears in each document. The dimensions of ***W ***are *M *× *N*, where *M *is the total number of words and *N *is the number of given documents. Each word count is normalized to compensate the differences in document lengths and overall counts of different words in the document collection [42].

Secondly, in order to recognize related or synonymous words and reduce the dimensions, singular value decomposition (SVD) is performed on the word-document matrix ***W***. Let *K *be the rank of ***W***. ***W ***is decomposed into three matrices by SVD:

(3) *W *= *USV*^*T*^

where ***U ***is left singular matrix with dimensions (*M *× *K*), ***S ***is diagonal matrix of singular values (*s*_1_≥ *s*_2 _≥ ... *s*_*K *_> 0) with dimensions (*K *× *K*), and ***V ***is right singular matrix with dimensions (*N *× *K*).

Thirdly, the dimensions of the solution are reduced by detecting the smaller singular values in ***S ***and ignoring the corresponding columns of ***U ***(rows of ***V***). Only the top *R *(*R *<< Min (*M*, *N*) dimensions for which the singular values in ***S ***are higher than a threshold are selected for further processing. Therefore, the dimensions of ***U***, ***S ***and ***V ***are reduced to *M *× *R*, *R *× *R *and *N *× *R*, leading to noise removal and data compression. Values of *R *ranging from 200 to 300 are typically used for information retrieval. In this study, the best results are achieved when *R *takes the value around 300. For a test document which is not in the training set, the unseen document is required to add to the original training set, so the latent semantic analysis model need to be recomputed. Because SVD is a computationally expensive process, it is not suitable to perform SVD every time for a new test document. The test document vector *t *can be approximated from the mathematical properties of the matrices ***U***, ***S ***and ***V ***as follow:

(4) *t *= *dU*

where *d *is the original vector of the test document, which is similar to the columns of the matrix ***W***.

Through performing SVD on the word-document matrix ***W***, the column vectors of ***W ***are projected onto the orthonormal basis which is formed by the row column vectors of the left singular matrix ***U***. The columns of ***SV***^*T *^give the coordinates of the vectors. Thus, the column vectors *Sv*_*j*_^*T *^or equivalently the row vectors *v*_*j*_*S *characterize the position of document *d*_*j *_in the *R *dimensions space. Each of the vector *v*_*j*_*S *refers to a document vector which is uniquely associated with the document in the training set.

In this study, Top-*n*-grams are treated as the "words" and the protein sequences are viewed as the "documents". Through collecting the weight of each word in the documents, the word-document matrix is constructed and then the latent semantic analysis is performed on the matrix to produce the latent semantic representation vectors of protein sequences, leading to noise-removal and smart description of protein sequences. The latent semantic representation vectors are then inputted into SVM to give the final results.

### Evaluation methodology

Two methods are used to evaluate the quality of the methods: the Receiver Operating Characteristic (ROC) scores and the ROC50 scores [48]. A ROC score is the normalized area under a curve that plots true positives against false positives for different classification thresholds. A score of 1 denotes perfect separation of positive samples from negative ones, whereas a score of 0 indicates that none of the sequences selected by the algorithm is positive. A ROC50 score is the area under the ROC curve up to the first 50 false positives.

### Statistical tests

To determine whether two methods have statistically different ROC or ROC50 scores on a particular benchmark, the Wilcoxon signed-rank test is used. The *p*-values are Bonferroni-corrected for multiple comparisons.

The chi-square algorithm is used to measure the correlation between a feature *t *and a protein family *c *and can be compared to the chi-square distribution with one degree of freedom to judge extremeness. The chi-square score of feature *t *relative to protein family *c *is defined to be:

(5) $$\chi^{2}(t,c)=\frac{N\times {\left(A\times D-C\times B\right)}^{2}}{\left(A+C)\times (B+D)\times (A+B)\times (C+D\right)}$$

where *A *is the number of times *t *and *c *co-occur, *B *is the number of times the *t *occurs without *c*, *C *is the number of times *c *occurs without *t*, *D *is the number of times neither *c *nor *t *occurs and *N *is the total number of protein sequences.

The chi-square statistic has a natural value of zero, if *t *and *c *are independent. The family-specific scores of each feature can be combined into an average chi-square score:

(6) $$\chi_{avg}^{2}(t)=\sum_{i=1}^{m}P_{r}(c_{i})\chi^{2}(t,c_{i})$$

where *P*_*r*_(*c*_*i*_) is the probability of protein family *c*_*i *_and *m *is the total number of protein families.

## Authors' contributions

BL carried out remote homology detection and fold recognition studies, participated in coding and drafting the manuscript. LL, QD and XW participated in the design of the study and performed the statistical analysis. XLW conceived of the study, and participated in its design and coordination. All authors read and approved the final manuscript.
